# Studies of mitochondrial genomics in *Salmo trutta fario*

**DOI:** 10.1080/23802359.2017.1331329

**Published:** 2017-05-26

**Authors:** Abolhasan Rezaei, Sheyda Akhshabi

**Affiliations:** Department of Genetics, School of Basic Science, Tonekabon Branch, Islamic Azad University, Tonekabon, Iran

**Keywords:** Salmo trutta fario, mitochondrial genomics

## Abstract

*Salmo trutta fario* population is an important species for aquaculture and livestock industry. Moreover, these species were used for studies on the molecular markers. Mitochondrial genomic is also beneficial for phylogenetic studies in salmonid species. They applied maternal traits for mitogenome, whereas paternal traits related to nuclear genomics. It will be genetically highly divergent indicating that they may represent distinct and potentially locally adapted gene pools (Apostolidis et al., 2011). Evolutionary history has been studied on the *Salmo* taxa such as brown trout, *Salmo salar*, *Salmo trutta* populations (Kottelat & Freyhof, 2007; Simonovic et al., 2007).

The evolutionary history of the *Salmo* taxa species including *Salmo salar* and *Salmo trutta* populations have been studied (Bernatchez 2001; Kottelat & Freyhof 2007). In the current study, 30 samples were collected from three major rivers in Iran: Cheshmeh Kileh, Ghasem Abad, and Jaj Roud. Total genomic DNA was isolated from muscle tissue (10 mg) taken from fresh specimens. The sample salmonids were caught in the Fall of 2015 and they were three years old. The left side was photographed and the right pectoral fin was clipped, tagged like the whole fish, and was subsequently fixed in 96% ethanol. DNA isolation was carried out following the phenol–chloroform protocol (Sambrook & Russel 2001). 10 ng of purified PCR product were sequenced. Mitogenomic study in *S.t. fario* is a new mitogenome sequenced for the first time in Iran and deposited in the GenBank (LC137015.1.) (Figure 1). The evolutionary history was inferred using the neighbour-joining Method (Saitou & Nei 1987)). Evolutionary analyses were conducted using MEGA7 (Kumar et al. 2016). Here, the optimal tree with the sum of branch length equal to 638.75 is shown. The tree is drawn to scale, with branch lengths in the same units as those of the evolutionary distances used to infer the phylogenetic tree, which were computed using the Maximum Composite Likelihood method (Tamura & Nei 1993). The analysis involved 50 nucleotide sequences. There were 16,622 positions in the final dataset. Results showed that a major diversity exists between *S.t. fario* and other salmonids like *Hucho taimen*, etc., *S.t. fario* and *S.t. caspius* are underlined in Figure 1 to show the close relationship observed between them.

**Figure 1.**
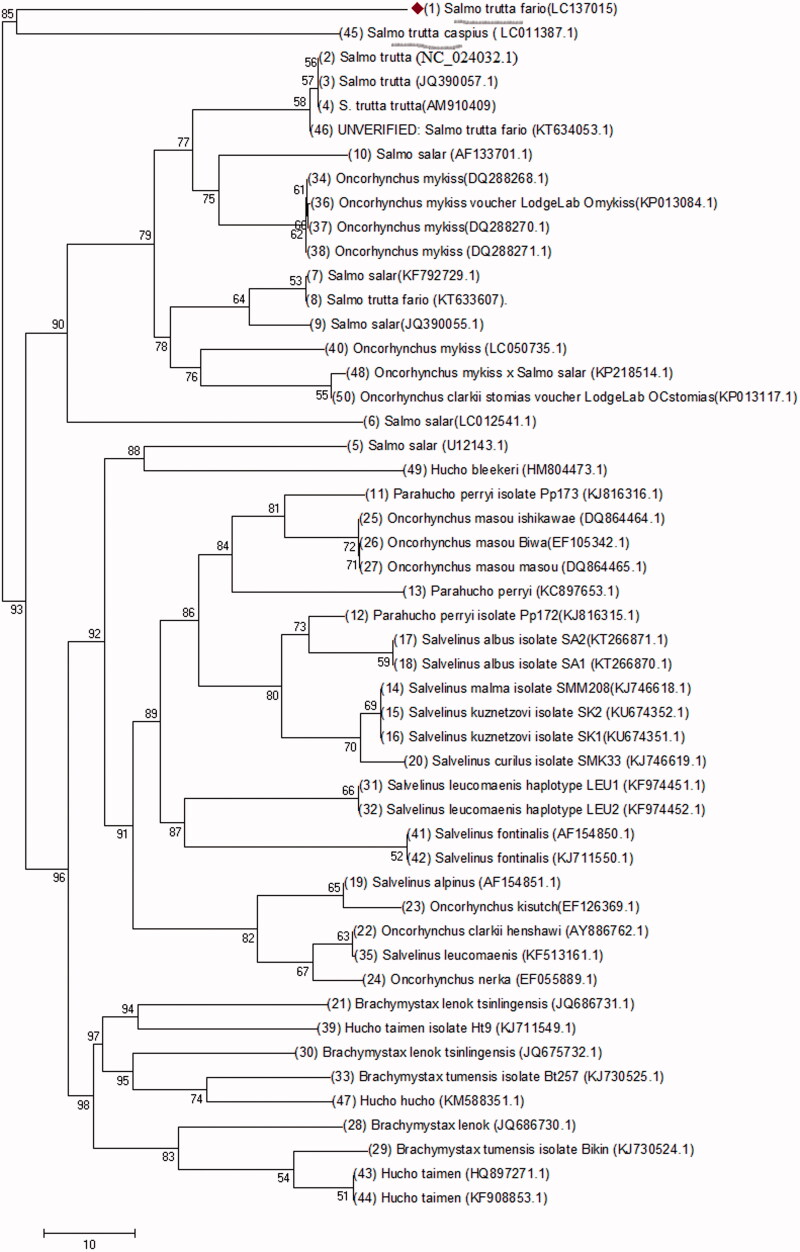
Phylogenetic analysis was done using an Omega program with 50 complete mitogenomes under family Salmonidae species. To investigate the phylogenetic analysis between mitogenomes of *S.t. fario* and other salmonids. *S.t. fario* and *S.t. caspius* are underlined.
